# The Influence of Emotional Health on the Activity Characteristics of the Elderly and the Selection of Environmental Quality Factors in Residential Areas

**DOI:** 10.3390/ijerph182312618

**Published:** 2021-11-30

**Authors:** Wen Zhong, Jian Suo, Xinxin Ren, Guopeng Li

**Affiliations:** Department of Architecture, School of Architecture and Fine Art, Dalian University of Technology, Dalian 116081, China; zwynlycg@163.com (W.Z.); renxinxin@dlut.edu.cn (X.R.); liguopeng@dlut.edu.cn (G.L.)

**Keywords:** residential area, the elderly, emotional health, activity characteristic, environmental quality factor

## Abstract

The environment in urban residential areas is the main field of daily activity for the elderly. Environmental renewal has played a significant role in improving residents’ quality of life and promoting physical and mental health. However, there is a general tendency that more attention has been focused on the environment during environmental renewal but not the residents. There is a continued lack of discussion on the emotional status of the elderly and its impact on outdoor activities. Based on the investigation of four types of typical residential environments in the Dalian residential area, a hierarchical linear model (HLM) has been constructed to reveal the influence of the emotional status of elderly persons on their activity characteristics and the selection of environmental quality factors. The results show that the distribution of older people with different emotional statuses varies among different residential types. The proportion of positive emotion is relatively high in the flat land in rows category, and the activity characteristics are significantly different under different emotional statuses. Among the four kinds of residential environments, which are (flat land in rows, flat land enclosed, slope, and mountainous) the environmental quality factors that have the highest impact on the emotional status of the elderly are greening quality (0.395), acoustic environment (0.167), environmental cleanliness (0.269), and greening quality (0.230), respectively. In the mountainous type, the impact of environmental quality factors on the emotional status of the elderly is the highest (39.7%), and the impact contributions of the other three environmental types are 23.3%, 8.9% and 20.1%, respectively. These research results provide helpful guidance for the scientific community about practical implementation of residential environmental renewal for the elderly.

## 1. Introduction

With the aggravation of ageing, the mental health problems of the elderly have aroused widespread concern in society. As the last stage of the life process, ageing is its most prominent feature, manifested in the degradation of physical function and the decline of cognitive ability and psychological changes at such different levels as emotion and personality [1]. Especially after retirement, the elderly are gradually separated from their original social roles and their living space and communication scope becomes narrowed. They easily fall into negative psychological states such as depression and anxiety, which reduce their quality of life and happiness index. Approximately 97% [2] of the elderly in China rely on the residential area and its service system to support their elderly care requirements [3]. Among them, the elderly population in residences built in the 1980s and 1990s is relatively concentrated, and the elderly population in many residences accounts for about one third [4]. In recent years, residential environment renewal carried out in various parts of China has improved residents’ quality of life. During residential renewal, reasonable allocation of environmental elements can provide nearby places for exercise and social networking for the elderly, and reduce their sense of loneliness and isolation [1]. After the outbreak of COVID-19, many older people chose to move into the neighbourhood space, which has a positive effect on improving their physical and mental health.

Studies report that the health status and life satisfaction of the elderly are highly related to emotional status [5,6]. Individuals with high emotional status often experience more positive emotions, while individuals with low emotional status often experience more negative emotions, which is not conducive to health. Many studies have investigated the relationship between environment and human emotion in Europe, America and other countries [7,8,9,10,11,12,13]. Guitea reported that residents’ dissatisfaction with social and entertainment facilities reduced their mental health levels [7]. In contrast, Carlson J.A. and others found that the built environment of the residential area has a synergistic effect with the social and psychological factors of elderly residents. A high-quality environment can promote the physical activities of the elderly [8]. Pacione analyzed the impact of personal and family characteristics such as age, income, education and physical health on mental health [9]. In addition, many research studies have considered the impact of green landscape on the emotions of the elderly [14,15,16,17,18]. Chen Chongxian found that different quality environments in built residences have different influencing factors on the emotion of the elderly [14]. Grahn and Zhou Suhong reported that the community environment affects residents’ way of thinking and behaviour, thus affecting their mental health [15,18].

More relevant research [6,7,8,9,10,11,12,13,14,15,16,17,18,19,20,21,22] mainly focuses on the macro and meso levels of the built environment. Recently, more attention has been paid to parks, green spaces and residential areas at a more micro level. The study area has also been selected from Mega cities like Chicago, Shanghai, and Guangzhou. The research on the emotional health and related impact of the elderly in the residential area is still insufficient, with limited guidance from the research results regarding the renewal of the residential environment. This study focuses on the impact of the emotional health of the elderly on their activity in the Dalian residential area, and on the selection of environmental quality elements in order to improve the outcomes and rationality of environmental quality renewal.

## 2. Materials and Methods

### 2.1. Study Design and Participants

The research data collection began in April 2021 and sampled four administrative locations in Dalian. As Dalian includes hilly landforms, in order to more comprehensively reflect the impact of the residential environment on the elderly, the study selected four distinct topographic environmental areas. Through measurement and observation, a total of 98 spaces between residential buildings were defined and categorized into four types: flat land in rows, flat land enclosed, sloped, and mountainous (Table 1). Flat land in rows and slope residences were generally outside the central urban area, while enclosed residences and mountainous residences were within the central urban area. Using typological methods, we subdivided the four types of residential environments studied into the 18 typical types with the highest proportions. Elderly residents over 60 years old who have lived in the study area for more than half a year and who could understand the questionnaire and provide responses were randomly selected. The purpose was introduced to the respondents during the survey, followed by simple questions about their age and residence time to determine whether they were eligible to participate. The survey interview was undertaken anonymously, with oral, informed consent obtained from each participant before commencing the study.

Additionally, in order to scientifically set the question option intervals, two sunny days were selected to record the behavioural trajectories of three residential samples of the elderly over 60 years old. This was done every half an hour in the early stage of the formal survey, with the behaviour trajectories of the two days superimposed (Table 2) [23] and the visual presentation of the trajectories adopted using ArcMap (version 10.7, ESRI, Redlands, CA, USA). The observation results showed that the number of older people who undertake outdoor activities was highest between 10:00–11:00 a.m. and 15:00–17:00. The activity duration was primarily in the range of 0.5 h–3 h, the activity frequency 1–2 times a day, and activity content varied according to the residence environment. The main activities included walking, sitting idle, basking in the sun, chatting, watching children, playing chess and cards, walking dogs, and sightseeing. A total of 312 questionnaires were distributed (85 from Wencuixuan Community, 81 from Shidaojie Community, 76 from Jixian Community, and 70 from Xigou Community). The questionnaire efficiency was 98%, with 306 valid questionnaires returned.

### 2.2. Measures

#### 2.2.1. Elderly Self-Assessment of Emotions and Health

The Newfoundland Memorial University Happiness Scale (MUNSH) was selected. This scale is based on the emotional status theory, that is, the status between positive and negative emotions. The design is divided into four dimensions: positive emotion (PA), negative emotion (NA), and positive Experience (PE) and Negative Experience (NE), for a total of 24 questions. An answer of “yes” to an item scores 2 points, “don’t know” scores 1 point and “no” scores 0 points. For item 19, an answer of “Current residence” scored 2 points and "Other residence" scored 0 points. An answer of “satisfied” on item 23 scored 2 points and “unsatisfied” scored 0 points. Total score=PA-NA+PE-NE, and the score range was −24~+24. In order to facilitate the calculation, the constant 24 is often added, for a score range of 0–48 (Table 3).

#### 2.2.2. Personal and Social Information of the Elderly

Controlling other factors that may affect the mental health outcomes of elderly residents is one of the focuses of this study. The controlling factors affecting the mental health of the elderly include individual, family and society. In the existing studies, Pinquar [24] believes that women may bear more psychological pressure than men in family life; Aldwin [25] found that the mental health of the elderly was significantly lower than that of the middle-aged and younger elderly. Some scholars [26,27,28,29,30,31] have found that annual income, education level, family structure, physical health status, neighborhood and family relations all have an impact on the mental health of the elderly. In addition, relevant research [32] shows that residential area and neighborhood environment also affect residents’ mental health. Based on the above research, the control variables selected in this paper included gender, age, education level, family annual income, physical health status, family structure, family and neighborhood relationship (Table 3).

#### 2.2.3. Evaluation of Residential Environment Elements and Collection of Physical Activity Rules

The selection of environmental elements considered the mainstream elements that appear in existing research, and at the same time referred to the relevant domestic policy standards and environmental characteristics of residential areas in Dalian. First, through a review of relevant domestic and foreign literature [33,34,35,36,37,38,39,40,41,42,43,44,45], a total of 57 environmental elements that affect the mental health and emotional perception of the elderly in the literature were extracted, similar items were merged, and elements that were compatible with the residential environment were retained based on field research. The three major categories of function, comfort, and place-specific spatial quality elements were summarized and sorted out [46]. Finally, referring to the six types of renovation indicators (water, air, comfort, fitness, humanities, and services) mentioned in the evaluation standard of residential health renovation [47], the elements were modified and supplemented, and a total of eleven residential environment elements were determined: activity and rest facilities, environmental cleanliness, spatial scale, greening quality, vegetation type, degree of sky openness, spatial scale, interface color, the wind environment, sunshine, and acoustic environment. To facilitate the understanding of the elderly, a descriptive explanation is adopted for each element in the questionnaire, ranging from “very inconsistent”, “relatively inconsistent”, “general”, “relatively consistent” to “very consistent” (Table 3).

### 2.3. Data Analysis

Data analysis was performed using SPSS (version 25.0, IBM, Armonk, NY, USA). Graphs and figures were generated by GraphPAD Prism version 8 (Graph PAD, San Diego, CA, USA). First, three multiple regression models were constructed, and multiple dimensional independent variable elements were included in the model. In all models, Model I added factors such as personal and family characteristics, health and social relations as control variables. Model II further added the activity characteristics of the elderly in order to analyze the impact of the activity characteristics of the elderly on emotional status. Finally, eleven environmental quality elements were added to model III. The purpose of this paper was to reveal the relationship between the emotional status of the elderly and the different dimensional factors.

## 3. Results

### 3.1. Sample Characteristics

A summary of the sociodemographic characteristics of the participants is presented in Table 4. A gender distribution of 42.8% males and 57.2% females made up the participants. Individuals’ ages were classified as either 60–69 (35.3%), 70–79 (45.1%), or over 80 years old, (19.6%). The highest education level of most participants was high school (43.1%), spouses (51%) dominated the family living structure, and the family annual income range was primarily 80,000–120,000 RMB (Table 4).

### 3.2. The Activity Characteristic of the Elderly in Different Emotional States and the Choice Tendency of Environmental Quality Factors

**Figure 1:**
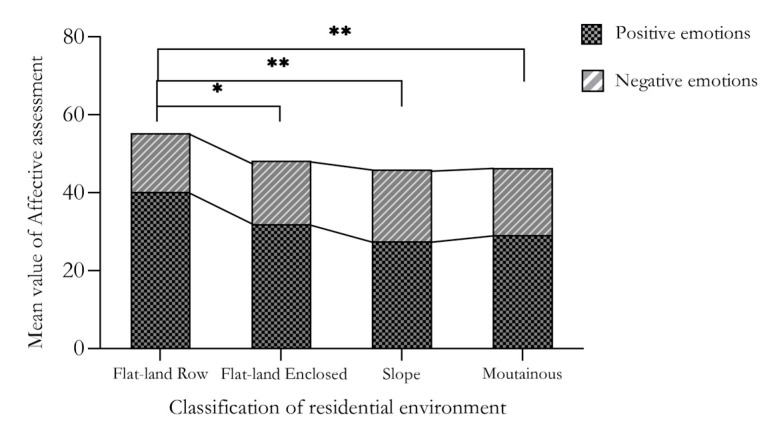
Emotional assessment of different types of residential environments. Note: *, ** indicate significant at the level of 0.05 and 0.01 respectively.

**Figure 2:**
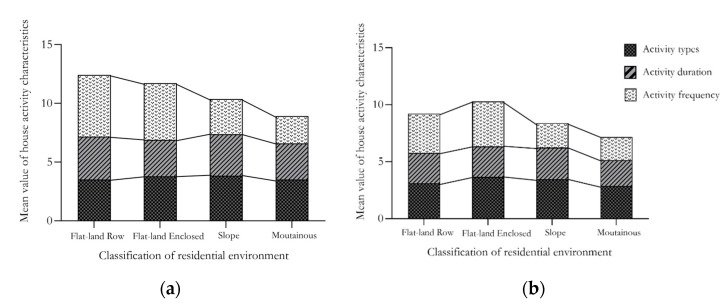
Activity characteristics of elderly people with different emotions. (**a**) positive emotion. (**b**) negative emotion.

**Figure 3:**
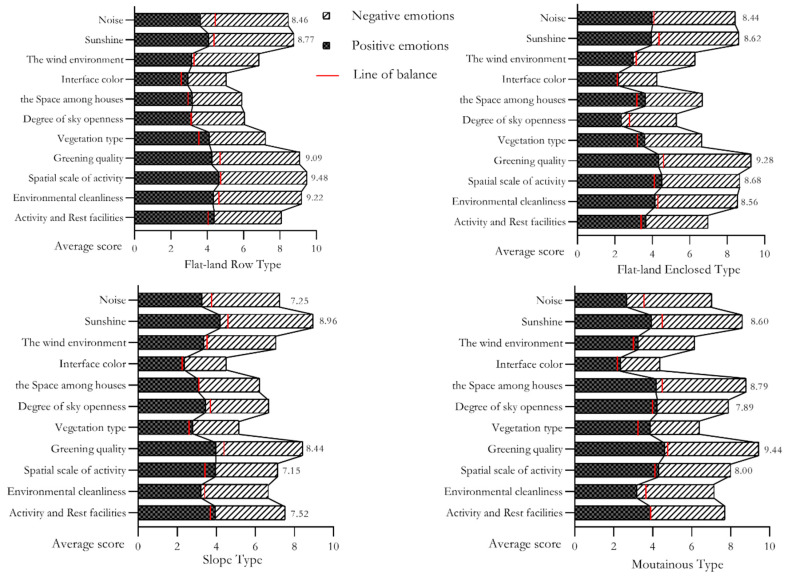
The elderly’s choice of environmental quality by two emotional types in different residential environments.

The participants were then divided into two emotional state groups according to their emotional status score: positive emotion (≥24 points) and negative emotion (<24 points). Firstly, the Kruskal–Wallis independent sample test was used to test the difference between each index group. The data analysis results summarized the differences in environmental quality selection, activity characteristics and emotional state of the elderly among the four residential environment types (Table 5 and Table 6). Under different living environments, there were significant differences between the participant groups with a positive emotion status, but there was no significant difference between the negative emotion groups. There were significant group differences in the characteristics of activities and various environmental quality indicators. Furthermore, the activity characteristics and environmental quality of the elderly in different emotional states were visualized and presented as a histogram (Figure 1, Figure 2 and Figure 3). As shown in (Figure 1), for flat land in rows-type residential participants, the average value of positive emotion was significantly higher than that of the other three types. The average value of negative emotion accounted for a relatively small proportion of the flat land in rows-type residents. There was little difference in the proportion of flat land enclosed, slope and mountainous-type residential participants.

The histogram in Figure 2a shows the differences in activity characteristics between the positive emotional groups. In different residential environments, the positive emotional group’s activity duration and activity frequency were relatively low. In the flat land row and flat land enclosed types, the activity types of the positive emotional group were more abundant than in the sloping and mountainous types. The trends for the group with negative emotions was similar (Figure 2b). In a horizontal comparison, the frequency, duration and type of activities in the positive emotional group were higher than those of the negative emotional group, inferring more active willingness and richer activity content for participants with a positive emotional status.

The selection of environmental quality by the elderly in positive or negative emotional states between different residential environments is examined in Figure 3. The elderly in the flat-land row type tended to choose the quality of activity space area (9.48), environmental cleanliness (9.22) and greening quality (9.09). Those in the negative emotional group focused more on the acoustic environment and the greening quality, while the positive emotional group paid more attention to the interface colour and vegetation types.

For the flat enclosed residential cohort, the quality factors most selected by the elderly were greening quality (9.28), activity space area (8.68), sunshine (8.62) and environmental cleanliness (8.56), which is similar to flat row residential environments. Sunshine and sky openness are the qualities that negative emotional participants focused on. The positive emotional group had a significantly greater tendency to choose spatial scale, vegetation types and activity space area than the negative emotional group.

For sloping-type residential participants, priority was given to three quality elements, including sunshine (8.96), greening quality (8.44) and activity and rest facilities (7.52). Among the mountainous cohort, the negative emotional group paid more attention to the quality of the acoustic environment, sunshine, and greening qualities. The positive emotional group was more inclined to choose activity space areas than the negative emotional group. The selection of other elements showed less significant differences.

For the mountainous-type cohort, the priority factors were greening quality (9.44), spatial scale (8.79) and sunshine (8.60). The positive emotional group were more inclined to choose vegetation types, while the negative emotional group paid more attention to the importance of acoustic environment and sunshine.

### 3.3. Impact Analysis

To further analyze the influence of the emotional status of the four types of homes on their activity rules and multi-factor selection, the study established three hierarchical linear models (HLM) (Table 7). According to the regression model’s collinearity and the independent tests’ residuals, the tolerance of all independent variables was greater than 0.2, and the Variance inflation factor (VIF) was less than 10. It can be deduced that no collinearity occurred in the independent variables of the model. The Durbin–Watson values were close to 2, indicating that the residuals were independent and met the statistical requirements.

In the flat land-type cohort, model IIa adjusted R^2^ = 0.506 (Sig. = 0.000). Compared with model Ia, adding the independent variables of the activity characteristics results in the R^2^ for the model increasing from 0.503 to 0.506. The effect of emotional status on activity characteristics increased by 0.3%, and the variable with a significant positive impact is the activity duration. The older the elderly with higher the emotional status level, the longer the activity duration. The Model IIIa adjusted R^2^ = 0.739 (Sig. = 0.000) compared with the Model IIa adjusted R^2^ increases by 0.233 units, showing that the participants’ emotional state influences the selection of environmental quality elements (23.3%). Model IIIa shows that emotional status is significantly positively correlated with the choice of environmental quality. There are three factors listed in descending order for the degree of influence: greening quality (β = 0.395), activity space area (β = 0.284), and acoustic environment (β = 0.263). Additionally, by comparing the adjusted R^2^ change values of models Ia, IIa and IIIa, it becomes apparent that IIa (0.3%) and IIIa (23.3%) are much smaller than the Ia (50.3%), indicating that the emotional status of the elderly in the flat land residential cohort affects the activity characteristics and activities. The degree of influence of the choice of environmental quality is relatively low.

In the flat land enclosed topology, model Ib adjusted R^2^ = 0.436 (Sig. = 0.000), and the emotional status of the elderly has an effect of 43.6%. With the addition of the independent variable of the activity characteristics in the residential environments, the model’s adjusted R^2^ increases from 0.436 to 0.497, indicating that the model’s emotional status on influencing the activity characteristics increased by 6.1%. The variables that have a positive correlation are activity frequency and duration; improving the emotional status of the elderly increases the frequency and duration of activities in the residential environments. Comparing model IIIb adjusted R^2^ = 0.586 (Sig. = 0.000) with model IIb, the adjusted R^2^ increases by 0.089 units, highlighting the effect of emotional status on participants. The ability to select factors affecting environmental quality is 8.9%. Model IIIb shows that emotional status significantly positively affects the selection of environmental quality. The degree of influence of the two elements is in descending order: acoustic environment (β = 0.167) and greening quality (β = 0.128). By comparing the adjusted R^2^ changes of models Ib, IIb and IIIb, it becomes apparent that IIb (6.1%) and IIIb (8.9%) are much smaller than the Ib (43.6%). The participants’ emotional status in the enclosed flat land-type had the lowest contribution to selecting environmental quality factors among the four residential environments.

In the sloping residential type, model Ic is adjusted to R^2^ = 0.588 (Sig. = 0.000). When variables of the activity characteristics are added, the adjusted R^2^ of the model increases to 0.623, and the impact effect increases by 3.5%. The variable with a positive impact is activity frequency. Model IIIc adjusted R^2^ = 0.824 (Sig. = 0.000); compared with Model IIc, adjusting R^2^ increases it by 0.201 units, inferring that the impact capacity is 20.1%. Model IIIc shows that emotional status significantly positively affects the selection of environmental quality. The degree of influence of the three elements is in descending order: environmental cleanliness (β = 0.269), acoustic environment (β = 0.233) and greening quality (β = 0.196). Comparing the adjusted R^2^ change values of models Ic, IIc, and IIIc, the contribution of emotional status to model Ic is much greater than that of IIc and IIIc, consistent with the first two types of residential environments.

In Mountainous residential types, model Id has an adjusted R^2^ = 0.388 (Sig. = 0.000). After adding the activity feature variables of the homes, the model adjusted R^2^ increased to 0.435, indicating that the effect of the model’s emotional status affecting activity features increased by 4.7%. With improved emotional status levels, the activity duration and activity type also increase significantly. Model IIId adjusted R^2^ = 0.832 (Sig. = 0.000) and adjusted the change of R^2^ to 0.397, highlighting the effect of emotional status on environmental quality elements in the model. The ability to select influence is 39.7%. Model IIId shows that the emotional status significantly positively affects the selection of environmental quality. There are four elements, in descending order of influence: greening quality (β = 0.230), sunshine (β = 0.229), spatial scale (β = 0.204) and activity rest facilities (β = 0.160). Comparing the adjusted R^2^ change values of models Id, IId and IIId, it becomes apparent that in Mountainous homes, the emotional status of the elderly affects the selection of environmental quality factors (39.7%) more than the control variable factors (38.8%). Such a trend is different from the first three types of residential environments.

## 4. Discussion

### 4.1. Different Activity Characteristics from Positive and Negative Emotions in Different Residential Environments, and Difference Analysis of the Selection of Environmental Quality Factors 

This study supports the original hypothesis and indicates that the elderly under different emotional states have different activity characteristics, as well as the selection tendency of environmental quality factors among the elderly by the type of residential environment. The study found that the positive emotions of older people who were active in flat residential environments were significantly higher than those of the other three types of residential environments. Negative emotions accounted for the smallest proportion in the flat residential type, and there was no significant difference in the proportions of the other three types. Behavior observations and questionnaire statistics were undertaken on the elderly who are active in the sample residential environments in order to explore the emotional differences in different residential environments. It was found that the frequency, duration and type of activities of the elderly in a positive emotional state were all higher than those of the negative state group. Participants within the negative emotional index subgroup had smaller activity durations and frequencies than the positive emotion group. However, the activity content of the elderly in the flat land type was much higher than that in the slope and mountainous types. Additionally, older people with negative emotions paid more attention to the acoustic environment and sunshine quality in terms of environmental quality. In contrast, the elderly with positive emotions paid more attention to interface colour and vegetation types.

### 4.2. Analysis of the Impact of the Emotional Status of the Elderly on the Choice of Environmental Quality among Different Types of Residential Environments

A hierarchical linear model was established to further study the relationship between the activity characteristics and environmental quality selection differences of the elderly in different residential environment types and with different emotional status levels. As the elderly cohort is a large group with genetic heterogeneity, health, cultural experiences and lifestyle [48,49], older people’s potential influence variables such as personal and family characteristics, health status, and neighbourhood relationships were controlled in the model. There is a significant relationship between the emotional status of the elderly, their choice of environmental quality, and activity characteristics among residential buildings; the relationship also varies with the type of residential environment. Some scholars have discussed the differences in impacts of the built environment from the macro, medium and micro perspectives, such as city [20], region [50], park green space [11,19], and residential area [14,51,52]. In contrast to the previous studies, the current study identifies the spatial scale in front of and behind residential buildings.

From the impact results, the contribution values of the emotional status of the elderly affected by the control variables were 50.3%, 43.6% and 58.8%, which were much higher than the variables such as activity characteristics and environmental quality. Unfortunately, few relevant studies focus on the built environment at the spatial scale. However, other studies based on alternate environmental scales [53,54] report that mental health affects the quality of the built environment far less than education, housing ownership, physical health and other factors, consistent with the results of this study. To some extent, such data infers that the built environment quality from macro (city) to micro (residential) environments and its attraction to the activities of the elderly are insufficient. In contrast, and different from the other three types, the mountainous residential type contributes significantly to the elderly’s emotional status. The selection of environmental quality elements is higher than the control variables. These findings are different from previous studies, indicating a high correlation between promoting mental health in the elderly and the selection of environmental quality elements in mountainous residential types.

The results infer that in the impact of emotional state on the selection of environmental quality elements, the greening quality is significantly affected by the emotional status of the elderly in all types of environments. The higher the emotional status level of the participants, the higher the requirements for greening quality. This shows that a high-quality green landscape is a quality commonly noted by the elderly during their daily activities. However, in different types of environments, their influence effects are different. The greening quality (0.395) is the highest in the flat row type and the lowest in the slope type (0.196). Carrus et al. [54] proposed that the location of greening in public spaces and the diversity of plants will lead to differences in the benefits of greening on people’s psychological well-being and recovery. The current study speculates that the differences in the growth forms of natural vegetation and artificial greening caused by terrain among different types of residential environments may be related to the great differences in the impact effects of greening quality. Furthermore, the length of activity time and participation in the greening landscape [7,19] will affect the degree of choice in greening quality for mental health.

Comparison of four types of residential environments shows obvious differences in the relationship between emotional states and environmental quality elements. The most critical environmental quality of flat and mountainous types is greening quality. A good acoustic environment is highly affected by emotional status in the flat row and sloping types, while low in the enclosed type. As Putrik et al. [55] proposed, one possible reason is that in the noise component of the built environment in the residential area, road traffic noise will significantly affect the residents’ mental health. The two sides of the flat row and sloping types are adjacent to the road, and are therefore greatly affected by traffic noise. The enclosed type is generally far away from motor vehicle roads and is less affected by traffic noise. In the sloping residential environment type, the factor with the highest outcome affected by emotional status is the cleanliness of the environment. This result is consistent with the findings of previous studies, which [56] reported that community untidiness (such as garbage and pet leavings) are associated with lower levels of community satisfaction and mental health. Notably, the three variables of vegetation type, interface colour and wind environment are not related to emotional status among the four types of residential environments. Some scholars [33] have studied the impact of street interface colour on psychological feelings such as security, comfort and pleasure through quantitative analysis, but did not address its impact on mental health.

### 4.3. Research Value and Deficiency

This study highlights the characteristics of the activity choices of different older people relative to their emotional status and the obvious differences in their choice of environmental factors among different residential environments. Therefore, more targeted and personalized renewal can be formulated, resulting in residential environments avoiding non-differentiated and superficial renewal methods. Overall, the emotional status level of the elderly groups should be improved by promoting the types and enthusiasm of outdoor activities of the elderly. Potential shortcomings of this study include: (1) the study area was limited to four typical residential areas in Dalian; the statistics on residential types may have limitations, and the number of samples and questionnaires may need to be expanded in future research; (2) The results about the relationship between emotional status, activity choice and environmental factor selection of the middle-aged and elderly during different seasons were not clear, and relevant research should continue in the future; (3) The research did not identify reasons for the large differences in emotional status between different types of homes, and future research is needed to clarify this finding; and (4) The selection criteria of influencing factors need to be further implemented in future research.

## 5. Conclusions

The current study analysed the emotional status of the elderly after the renewal of a low-standard residential environment in China. The aim was to explore the relationship between the emotional status of the elderly in different types of residential environments and their choice of activity characteristics and environmental quality factors. By constructing a hierarchical linear model, this investigation analysed the influence and contribution of the emotional status of elderly residents to different dimensions of variables, and the effectiveness and reliability of the model were fully validated. The innovative conclusions are as follows: 

Firstly, the level of positive emotion in the elderly living in the flat land in rows type of accommodation is significantly higher than that of the elderly living in the other three types. The activity frequency, duration and type of activities undertaken by the elderly experiencing a positive emotional state is higher than for those in the negative emotional states. From the perspective of residence types, the activity types of the elderly in flat residences are more abundant than those in sloping and mountainous-type residences. Secondly, in the four types of residential environments, flat land in rows, flat land enclosed, slope, and mountainous, the effects of emotional status on the activity characteristics of the elderly are 0.3%, 6.1%, 3.5% and 4.7%, respectively. Among the four residential environments, the environmental quality factors with the highest effect on the emotional status of the elderly are greening quality (0.395), acoustic environment (0.167), environmental cleanliness (0.269) and greening quality (0.230). Finally, the study found that among the Mountainous residential cohort, emotional status had the highest contribution to selecting environmental quality factors (39.7%). The contributions of the other three environments were 23.3%, 8.9% and 20.1%, respectively.

In general, these research results highlight the significant impacts of environmental quality elements on the emotional health of the elderly in different residential environment types. Practical guidelines for reducing the impact of adverse environmental quality factors such as degree of sky openness, spatial scale, and sunshine quality in residential renewal design can now be proposed, enabling the optimization of existing environmental governance methods. Equally, it provides an essential resource for residential property services and management.

## Figures and Tables

**Table 1:** Four types of typical residential areas in Dalian.

X Community			J Community		
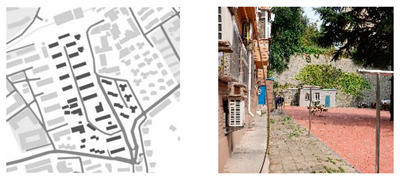			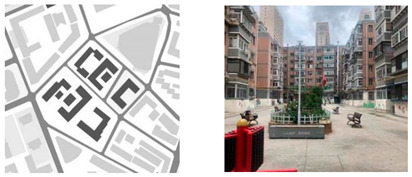		
Construction year	Space type	Number of households	Construction year	Space type	Number of households
1979–1988	Flat-land Row Type	672	1982–2000	Flat-land Enclosed Type	2797
**W Community**			**S Community**		
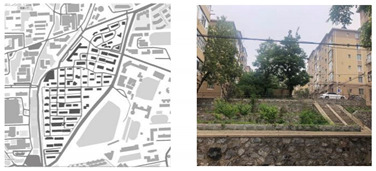			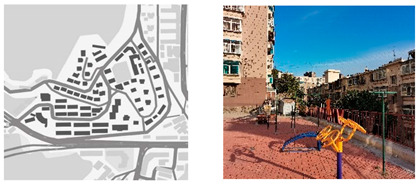		
Construction year	Space type	Number of households	Construction year	Space type	Number of households
1980–1989	Slope Type	984	1986–1987	Mountainous Type	3577

**Table 2:** Characteristics of residential activities of the elderly on sunny days (overlapping two-day data 8:00–18:00).

Sample 1	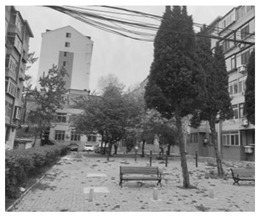	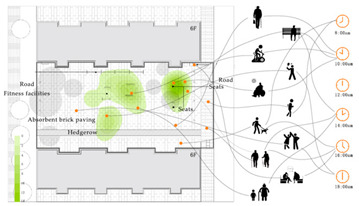
Sample2	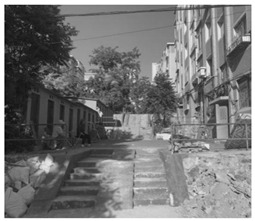	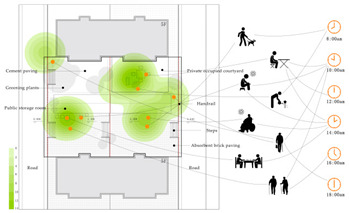
Sample 3	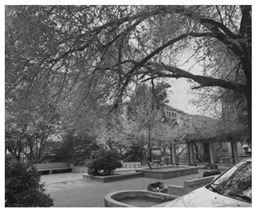	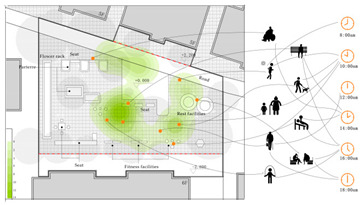

**Table 3:** Index system.

Index System		Influence Variable	Variable Level and Assignment
The basic attributes of individual society		gender	2 Categories (1 = male; 2 = female)
		age	3 Levels (60–69 years old, 70–79 years old, over 80 years old)
		education level	5 Levels (primary school or below, junior high school, senior high school or vocational high school, Associate degree, Bachelor’s Degree)
		Family annual income	6 Levels (below 10,000, 10,000 to 30,000, 30,000 to 80,000, 80,000 to 120,000, 120,000 to 200,000, more than 200,000)
		Housing area	3 Levels (below 50 m^2^, 50–80 m^2^, above 80 m^2^)
		Family structure	4 Categories (living alone, with spouse, second generation, third generation)
Individual social relations		Physical condition	5 levels (assign 1 = very bad~5 = Very good)
		Family relationships	5 levels (assign 1 = very bad~5 = Very good)
		neighborhood	5 levels (assign 1 = very bad~5 = Very good)
Activity characteristics		Activity frequency	4 levels (1–2 times, 3–4 times, 5–6 times, 7 times or more)
		Activity duration	5 Levels (less than 0.5 h, 0.5–1 h, 1–2 h, 2–3 h, 3 h and above)
		The activity type	Record the content of the activity, and count the number of types
Multiple quality variables in the residential environment	Functional Quality	Activity and Rest facilities	5 levels (1 = very inconsistent~5 = very in conformity)
		Environmental cleanliness	5 levels (1 = very inconsistent~5 = very in conformity)
		Spatial scale	5 levels (1 = very inconsistent~5 = very in conformity)
	Place Quality	Greening quality	5 levels (1 = very inconsistent~5 = very in conformity)
		Vegetation type	5 levels (1 = very inconsistent~5 = very in conformity)
		Degree of sky openness	5 levels (1 = very inconsistent~5 = very in conformity)
		Spatial scale	5 levels (1 = very inconsistent~5 = very in conformity)
		Interface color	5 levels (1 = very inconsistent~5 = very in conformity)
	Comfort Quality	The wind environment	5 levels (1 = very inconsistent~5 = very in conformity)
		Sunshine	5 levels (1 = very inconsistent~5 = very in conformity)
		Acoustic environment	5 levels (1 = very inconsistent~5 = very in conformity)
emotion healthy		Emotional status	MUNSH Happiness Scale, a total of 24 questions, total score = PA-NA+PE-NE. Score range −24~+24

**Table 4:** Basic information statistics of the elderly interviewed (n = 306).

Characteristic	Category	N (%)
Sex	Male	131 (42.8%)
	Female	175 (57.2%)
Age	60–69	108 (35.3%)
	70–79	138 (45.1%)
	≥80	60 (19.6%)
Education	Primary school or below	62 (20.3%)
	Junior high school	69 (22.5%)
	High school or vocational high school	132 (43.1%)
	Associate degree	33 (10.8%)
	Bachelor’s Degree	10 (3.3%)
Family living structure	Living alone	85 (27.8%)
	With spouse	156 (51.0%)
	Two generations	49 (16.0%)
	Three generations	16 (5.2%)
Annual household income(RMB)	10,000–30,000	46 (15.1%)
	30,000–80,000	161 (52.6%)
	80,000–120,000	69 (22.5%)
	120,000–200,000	24 (7.8%)
	≥200,000	6 (2.0%)

**Table 5:** Comparison of emotional state and activity characteristics among different types of residential environments.

Individual Situation of the Elderly		Residential Environment Type	Mean	Differences between Groups
Emotional state	Positive emotion	Flat-land in rows	40.17	0.001 **
		Flat-land enclosed	31.98	
		Slope	27.48	
		Mountainous	29.07	
	Negative emotion	Flat-land in rows	15.04	0.635
		Flat-land enclosed	16.28	
		Slope	18.43	
		Mountainous	17.20	
Activity characteristics	Activity frequency	Flat-land in rows	3.31	0.016 *
		Flat-land enclosed	3.74	
		Slope	3.66	
		Mountainous	3.20	
	Activity duration	Flat-land in rows	3.15	0.022 *
		Flat-land enclosed	2.89	
		Slope	3.15	
		Mountainous	2.68	
	activity type	Flat-land in rows	4.36	0.000 **
		Flat-land enclosed	4.39	
		Slope	2.58	
		Mountainous	2.17	

Note: *, ** indicate significant at the level of 0.05 and 0.01 respectively.

**Table 6:** Comparison of Environmental quality assessment among different types of residential environments.

Residential Environment Type	Environmental Factor Index	Mean	Differences between Groups	Environmental Factor Index	Mean	Differences between Groups
Flat-land in rows	Activity and Rest facilities	3.76	0.000 **	Spatial scale	4.56	0.000 **
Flat-land enclosed		4.64			3.04	
Slope		2.67			4.15	
Mountainous		2.19			3.23	
Flat-land in rows	Environmental cleanliness	4.28	0.000 **	Interface color	3.34	0.000 **
Flat-land enclosed		3.2			3.63	
Slope		3.48			4.10	
Mountainous		3.19			3.98	
Flat-land in rows	Activity scale	4.34	0.000 **	Wind environment	4.31	0.000 **
Flat-land enclosed		3.93			3.32	
Slope		3.10			4.23	
Mountainous		3.16			3.46	
Flat-land in rows	Greening quality	3.16	0.000 **	Sunshine	4.41	0.000 **
Flat-land enclosed		2.46			3.71	
Slope		3.36			4.11	
Mountainous		4.08			3.28	
Flat-land in rows	Vegetation type	2.98	0.000 **	Acoustic environment	3.85	0.000 **
Flat-land enclosed		2.43			3.07	
Slope		4.33			4.36	
Mountainous		4.56			4.25	
Flat-land in rows	Degree of sky openness	4.41	0.000 **			
Flat-land enclosed		3.17				
Slope		3.93				
Mountainous		2.83				

Note: ** indicate significant at the level of 0.01.

**Table 7:** Multi-factor hierarchical regression analysis of the same type of residential environments.

	Influence Factors		Flat-Land in Rows Type			Flat-Land Enclosed Type			Slope Type			Mountainous Type		
			Model Ia	Model IIa	Model IIIa	Model Ib	Model IIb	Model IIIb	Model Ic	Model IIc	Model IIIc	Model Id	Model IId	Model IIId
Basic information of individual society	gender		0.086	0.011	−0.061	0.051	−0.133	−0.754	0.040	0.014	−0.049	0.078	0.052	−0.055
	age		0.072	0.116	0.102	0.074	−0.048	0.056	−0.045	−0.054	−0.041	−0.038	0.023	−0.033
	education level		0.158	0.128	0.067	0.172 *	0.143 *	−0.171	0.009	0.035	0.070	−0.083	−0.012	−0.037
	Family income		0.314 **	0.006	0.014	0.291 **	0.180 *	−1.764	0.179	0.103	0.077	0.330 **	0.074	−0.028
	Housing area		0.078	0.084	0.077	−0.001	0.122	0.380	−0.115	0.182 *	−0.066	0.003	−0.052	−0.040
	Physical condition		0.262 *	0.125	0.098	0.321 **	0.207 **	1.443	0.317 **	0.237 *	−0.093	0.481 **	0.079	0.041
	Family structure	With spouse	0.378 **	0.179	0.216 *	0.258 **	0.204 **	0.247 **	0.293 **	0.247 **	0.204 *	0.002	0.160	0.114
		Two generations	0.181	0.151	0.048	0.247 **	0.199 **	0.219 **	0.248 *	0.239 **	0.116	0.173 *	0.002	0.096
		Three generations	0.113	0.138	−0.036	0.049	0.080	0.048	0.072	0.123	0.083	0.009	−0.055	0.073
		Living alone	0	0	0	0	0	0	0	0	0	0	0	0
Social and family relations	Family relationships		0.108	0.099	0.077	0.125	0.074	−0.464	0.330 **	0.255 *	0.074	0.101	0.098	−0.005
	Neighborhood relationships		0.024	0.003	−0.030	0.032	0.005	−0.326	−0.150	−0.095	−0.100	0.029	0.022	−0.008
Activities Features	Activity frequency			0.212 *	−0.046		0.920 **	0.215 **		0.245 *	0.190 *		0.183	0.027
	Activity duration			0.288 **	0.225 **		−0.118	0.198 **		0.138	0.106		0.352 **	0.154 *
	type of activity			0.170	0.046		−0.031	0.124		0.238 *	0.100		0.257 **	0.257 **
Environmental multi-quality elements	Activity and Rest facilities				−0.078			0.137 *			−0.027			0.160 *
	Environmental cleanliness				0.075			0.084			0.269 *			−0.097
	Activity space area				0.284 **			0.035			0.111			0.209
	Greening quality				0.395 **			0.217 *			0.196 *			0.230 **
	Vegetation type				0.138			0.094			0.149			0.103
	Degree of sky openness				−0.005			−0.095			0.149			0.065
	Spatial scale				0.065			0.173			−0.017			0.204 *
	Interface color				0.150			0.024			0.002			0.092
	Wind environment				0.094			0.002			0.083			0.088
	Sunshine				−0.130			0.018			−0.083			0.229 *
	Acoustic environment				0.263 *			0.225 **			0.233 **			−0.070
	Adjusted R^2^		0.503	0.506	0.739	0.436	0.497	0.586	0.588	0.623	0.824	0.388	0.435	0.832
	F		2.389	2.875	3.080	3.569	3.746	4.492	3.135	4.740	4.805	4.064	3.223	3.776
	Sig.		0.000 **	0.000 **	0.000 **	0.000 **	0.000 **	0.000 **	0.000 **	0.000 **	0.001 **	0.000 **	0.000 **	0.000 **

Note: *, ** indicate significant at the level of 0.05 and 0.01 respectively.

## Data Availability

The data are not publicly available due to ongoing data analysis for subsequent research manuscripts.

## References

[1] Kowitt S.D., Aiello A.E., Callahan L.F. (2020). Associations among neighborhood poverty, perceived neighborhood environment, and depressed mood are mediated by physical activity, perceived individual control, and loneliness. Health Place.

[2] Notice of the Municipal Government on Printing and Distributing the 12th Five Year Plan for the Development of Civil Affairs in Shanghai. https://www.shanghai.gov.cn/nw29273/20200820/0001-29273_31711.html.

[3] 2021 Community Home-Based Elderly Care Status and Future Trend Report. https://research.ke.com/121/ArticleDetail?id=450.

[4] Tu H.J., Lu X.J. (2020). Rethinking of "9073" Pension Service Structure—Based on Survey on Decision-making of Architectural Planning Group of Old Residential Quarters in Shanghai. Hous. Sci..

[5] Zhang J.M., Liu Z.Y., Zhu Y.S. (2016). The mediating effect of affect balance in the relationship between health condition and life satisfaction in long-lived elders: The application of structural equation modeling. Fudan Univ. J. Med Sci..

[6] Leslie E., Cerin E. (2008). Are perceptions of the local environment related to neighbourhood satisfaction and mental health in adults?. Prev. Med..

[7] Guitea H.F., Clark C., Ackrill G. (2006). The Impact of the Physical and Urban Environment on Mental Well-Being. Public Health.

[8] Carlson J.A., Sallis J.F., Conway T.L. (2012). Interactions between psychosocial and built environment factors in explaining older adults’ physical activity. Prev. Med..

[9] Pacione M. (2003). Urban environmental quality and human wellbeing—A social geographic perspective. Landsc. Urban Plan..

[10] Koohsari M.J., Badland H., Mavoa S., Villanueva K., Francis J., Hooper P., Owen N., Giles-Corti B. (2018). Are Public Open Space Attributes Associated with Walking and Depression. Cities.

[11] Sturm R., Cohen D.A. (2004). Suburban Sprawl and Physical and Mental health. Public Health.

[12] Nutsford D., Pearson A.L., Kingham S. (2013). An ecological study investigating the association between access to urban green space and mental health. Public Health.

[13] Gandelman N., Piani G., Ferre Z. (2012). Neighborhood Determinants of Quality of Life. Happiness Stud..

[14] Chen C.X., Luo W.J., Kang N. (2020). Study on the Impact of Residential Outdoor Environments on Mood in the Elderly in Guangzhou, China. Sustainability.

[15] Zhou S.H., He J.M. (2017). Effects of spatial-temporal constraints of suburban residents on fitness activities to mental health in the context of rapid suburbanization: A case study in Guangzhou, China. Prog. Geogr..

[16] Chen Z. (2018). Assessing the Impact of High-density High-heterogeneity Urban District Landscape on Psychological Health and Optimizing via Evidence-based Design. Landsc. Archit..

[17] Dong Y., Li Z., Dong W. (2021). Relationship Between Green Space Perception of Communities and Residents’ Stress Level: A Case Study of 12 Communities in Harbin. Landsc. Archit..

[18] Grahn P., Stigsdotter U.A., Grahn P. (2003). Landscape planning and stress. Urban For. Urban Green..

[19] Lachowycz K., Jones A.P. (2013). Towards a better understanding of the relationship between greenspace and health: Development of a theoretical framework. Landsc. Urban Plan..

[20] Wu Y.T., Prina A.M., Jones A., Barnes L.E., Matthews F.E., Brayne C., CFAS M. (2017). Micro-Scale Environment and Mental Health in Later Life: Results from the Cognitive Function and Aging Study II. J. Affect. Disord..

[21] Weich S., Blanchard M., Prince M., Burton E., Erens B., Sproston K. (2002). Mental health and the built environment: Cross-sectional survey of individual and contextual risk factors for depression. Br. J. Psychiatry J. Ment. Sci..

[22] Araya R., Montgomery A., Rojas G., Fritsch R., Solis J., Signorelli A., Lewis G. (2007). Common mental disorders and the built environment in santiago, chile. Br. J. Psychiatry.

[23] Suo J., Zhong W. Study on the influence of residential environment on the activities of the elderly in existing residential areas—Taking Dalian as an example. S. Archit..

[24] Pinquar M., Sorensen S. (2001). Gender Differences in Self-Concept and Psychological Well-Being in Old Age: A Meta-Analysis. J. Gerontol..

[25] Aldwin C.M. (1991). Does age affect the stress and coping process? Implications of age differences in perceived control. J. Gerontol..

[26] Buckner J.C. (1988). The development of an instrument to measure neighborhood cohesion. Am. J. Community Psychol..

[27] Dolan P., Peasgood T., White M. (2008). Do we really know what makes us happy? A review of the economic literature on the factors associated with subjective well-being. J. Econ. Psychol..

[28] Ewing R., Handy S. (2009). Measuring the Unmeasurable: Urban Design Qualities Related to Walkability. J. Urban Des..

[29] Evans G.W. (2003). The built environment and mental health. J. Urban Health-Bull. N. Y. Acad. Med..

[30] Blanchflower D.G., Oswald A.J. (2004). Well-Being Over Time in Britain and the USA. Soc. Sci. Electron. Publ..

[31] Abraham A., Sommerhalder K., Abel T. (2010). Landscape and well-being: A scoping study on the health-promoting impact of outdoor environments. Int. J. Public Health.

[32] Dempsey N. (2008). Does quality of the built environment affect social cohesion? Proceedings of the ICE. Urban Des. Plan..

[33] Xu L.Q., Meng R.X., Chen Z. (2017). Fascinating Streets: The Impact of Building Facades and Green View. Landsc. Archit..

[34] Wandersman A., Nation M. (1998). Urban neighborhoods and mental health: Psychological contributions to understanding toxicity, resilience, and interventions. Am. Psychol..

[35] Farrell S.J., Aubry T., Coulombe D. (2004). Neighborhoods and neighbors: Do they contribute to personal well-being?. J. Community Psychol..

[36] Sullivan W.C., Kuo F.E., Depooter S.F. (2004). The fruit of urban nature vital neighborhood spaces. Environ. Behav..

[37] Beyer K.M., Kaltenbach A., Szabo A., Bogar S., Nieto F.J., Malecki K.M. (2014). Exposure to neighborhood green space and mental health: Evidence from the survey of the health of Wisconsin. Int. J. Environ. Res. Public Health.

[38] Pope D., Tisdall R., Middleton J., Verma A., van Ameijden E., Birt C., Macherianakis A., Bruce N.G. (2015). Quality of and access to green space in relation to psychological distress: Results from a population-based cross-sectional study as part of the EURO-URHIS 2 project. Eur. J. Public Health.

[39] Gehl J.A. (1989). changing street life in a changing society. Places A Q. J. Environ. Des..

[40] Smith R.J., Lehning A.J., Dunkle R.E. (2013). Conceptualizing age-friendly community characteristics in a sample of urban elders: An exploratory factor analysis. J. Gerontol. Soc. Work.

[41] Vallée J., Cadot E., Roustit C., Parizot I., Chauvin P. (2011). The Role of Daily Mobility in Mental Health Inequalities: The Interactive Influence of Activity Space and Neighbou rhood of Residence on Depression. Soc. Sci. Med..

[42] Wong M., Yu R., Woo J. (2017). Effects of perceived neighbourhood environments on self-rated health among community-dwelling older Chinese. Int. J. Environ. Res. Public Health.

[43] Fu Q. (2018). Communal Space and Depression: A Structural-Equation Analysis of Relational and Psycho-Spatial Pathways. Health Place.

[44] Sugiyama T., Leslie E., Giles-Corti B., Owen N. (2008). Associations of neighbourhood greenness with physical and mental health: Do walking, social coherence and local social interaction explain the relationships?. J. Epidemiol. Community Health.

[45] Schinasi L.H., Benmarhnia T., Roos A. (2018). Modification of the association between high ambient temperature and health by urban microclimate indicators: A systematic review and meta-analysis. Environ. Res..

[46] Fan Y., Li Z.B., Dong L. (2018). Connotation and renewal mode of architectural quality of existing residential areas in China. New Build..

[47] China Academy of Building Sciences Co., Ltd (2020). Assessment Standard for Healthy Retrofitting of Existing Residential Area.

[48] Aging in Times of the COVID-19 Pandemic: Avoiding Ageism and Fostering Intergenerational Solidarity.

[49] Sabrina C., Francesca G. (2021). Older People’s Lived Perspectives of Social Isolation during the First Wave of the COVID-19 Pandemic in Italy. Int. J. Environ. Res. Public Health.

[50] Tridib B., William C.B. (2018). Beyond Neighborhood Units—Living Environment and Public Policy.

[51] Bond L., Kearns A., Mason P., Tannahill C., Egan M., Whitely E. (2012). Exploring the relationships between housing, neighbourhoods and mental wellbeing for residents of deprived areas. BMC Public Health.

[52] Dunstan F., Fone D.L., Glickman M., Palmer S. (2013). Objectively Measured Residential Environment and Self-Reported Health: A Multilevel Analysis of UK Census Data. PLoS ONE.

[53] Qiu Y.Z., Chen H.S., Li Z.G. (2019). Exploring neighborhood environmental effects on mental health: A case study in Guangzhou, China. Prog. Geogr..

[54] Carrus G., Scopelliti M., Lafortezza R. (2015). Go greener, feel better? The positive effects of biodiversity on the well-being of individuals visiting urban and peri-urban green areas. Landsc. Urban Plan..

[55] Putrik P., Nanne K., Mujakovic S. (2015). Living environment matters: Relationships between neighborhood characteristics and health of the residents in a Dutch municipality. J. Community Health.

[56] Gale C.R., Dennison E.M., Cooper C., Sayer A.A. (2011). Neighbourhood environment and positive mental health in older people: The Hertfordshire Cohort Study. Health Place.

